# Operative Treatment of Adult Pyogenic Spondylodiscitis: A Retrospective Study of 32 Cases

**DOI:** 10.7759/cureus.14820

**Published:** 2021-05-03

**Authors:** Frideriki Poutoglidou, Dimitrios Metaxiotis, Panagiotis Saloupis, Anastasios Mpeletsiotis

**Affiliations:** 1 Orthopaedic Department, Papageorgiou General Hospital of Thessaloniki, Thessaloniki, GRC; 2 Orthopaedic Department, Hippokratio General Hospital of Thessaloniki, Thessaloniki, GRC

**Keywords:** spondylodiscitis, vertebral osteomyelitis, infection, surgery

## Abstract

Background

Spondylodiscitis is a potentially life-threatening infection that imposes a significant financial burden on healthcare systems. Current reports suggest an increase in the incidence of spondylodiscitis, which could be attributed to the aging population and the growing rates of drug abuse. This study aims to evaluate the safety and effectiveness of surgical treatment of spondylodiscitis.

Materials and methods

Thirty-two cases diagnosed with spondylodiscitis and treated operatively between 2010 and 2015 were enrolled in this study. Indications for surgery were progressive neurologic involvement, progressive spinal deformity or instability, impending fracture, epidural abscess, and poor response to antibiotics. Patients underwent a single-stage procedure. A combined anterior and posterior approach was used in 28 of the patients. In 20 patients, a titanium mesh cage was used for reconstruction. The mean follow-up was 5.6 years.

Results

There were 18 males and 14 females. The mean age of the patients was 68.4 years (range 56-78). The cervical spine was affected in two cases (6.3%), the thoracic spine in 12 cases (37.5%), and the lumbar spine in 18 cases (56.3%). The most commonly isolated microorganisms were *Staphylococcus aureus *and *Escherichia coli. *There was neither mortality nor severe complications. Fusion was achieved in all the patients. There was complete resolution of the neurologic deficits that were recorded preoperatively. No signs of recurrent or residual infection were recorded until the last follow-up.

Conclusions

Our data suggest that early detection and surgical intervention of spondylodiskitis is associated with favorable outcomes.

## Introduction

Spondylodiscitis (SD) refers to the infection of the intervertebral disk by a pathogen with secondary infection of the adjacent vertebral bodies. SD is a rare disease corresponding to 3%-5% of all the cases of osteomyelitis [1], with a varying incidence from one per 100.000/year to 1 per 250.000/year [1-2]. SD predominantly affects elderly patients with comorbidities such as diabetes mellitus, immunodeficiency, or malignancies [3]. The longer life expectancy, intravenous drug abuse, and higher diagnostic efficacy obtained by magnetic resonance imaging (MRI) could be the cause of the increasing frequency of SD reported nowadays [4-5].

The diagnosis of SD is established based on clinical and imaging features and laboratory investigations [6]. The most common symptoms are pain, fever, night sweats, and malaise with or without neurologic involvement [7]. As far as the imaging modalities are concerned, MRI is the gold standard for the diagnosis of SD [8]. The laboratory workup usually reveals elevated erythrocyte sedimentation rate (ESR), elevated C-reactive protein (CRP), and leukocytosis [6]. The pathogen involved is identified by blood culture or computed tomography (CT)-guided biopsy. Recently applied molecular techniques have increased sensitivity and specificity and have decreased the time to diagnosis [9].

The Initial management of SD includes bracing and long-term, pathogen-specific antibiotics. However, surgical treatment must be considered in patients with progressive neurologic deficits, progressive spinal deformity, or instability, in refractory cases, and antibiotic-resistant sepsis [10]. In this study, we report our long-term outcomes of operative treatment of 32 patients affected by SD. Also, we present the microorganism epidemiology of those cases and their neurological status pre- and postoperatively.

## Materials and methods

Patient population and selection

The present study received an exemption determination by the hospital’s Ethics Committee. Thirty-two patients diagnosed with SD and treated operatively between 2010 and 2015 were enrolled in this study. Records of each patient were collected using an electronic database. The diagnosis of SD was established based on the clinical history, physical examination, laboratory workup (including ESR, CRP, white blood cell count (WBC)), and radiological examinations (radiographs, MRI with gadolinium contrast, CT, or Technetium Tc99m bone scan). Blood cultures were obtained from all the patients, even if afebrile, and a CT-guided biopsy was performed when blood cultures were negative.

Initially, all the patients had no indication for surgery and were treated with intravenous pathogen-specific antibiotics and bracing (rigid cervicothoracic/thoracolumbar orthosis based on the segment involved). The causative microorganism had been identified from the blood cultures or CT-guided biopsy. Indications for surgery were progressive neurologic involvement, progressive spinal deformity or instability, impending fracture, epidural abscess, and poor response to antibiotics. The mean follow-up was 5.6 years (range 3.5-6.5 years).

Surgical technique

**Figure 1:**
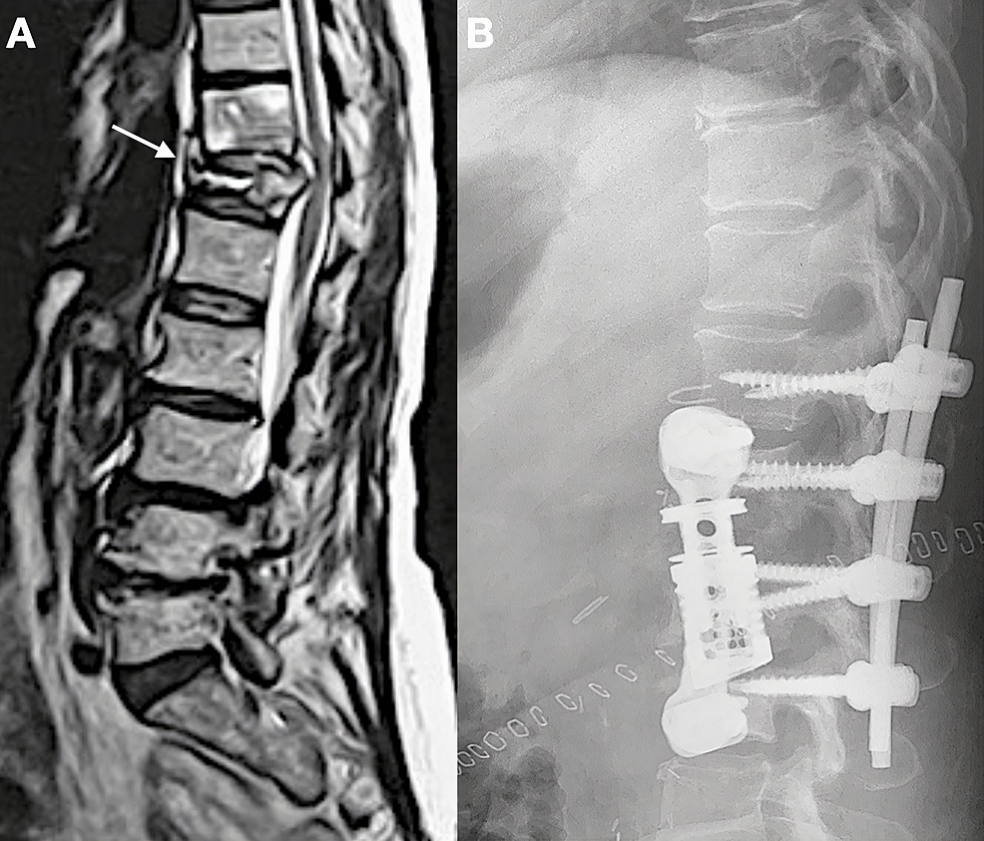
A. Preoperative sagittal T2-weighted image of the lumbar and thoracic spine, showing T12 spondylodiskitis (arrow); B. Postoperative lateral radiograph after the introduction of a titanium mesh cage and posterior instrumentation

**Figure 2:**
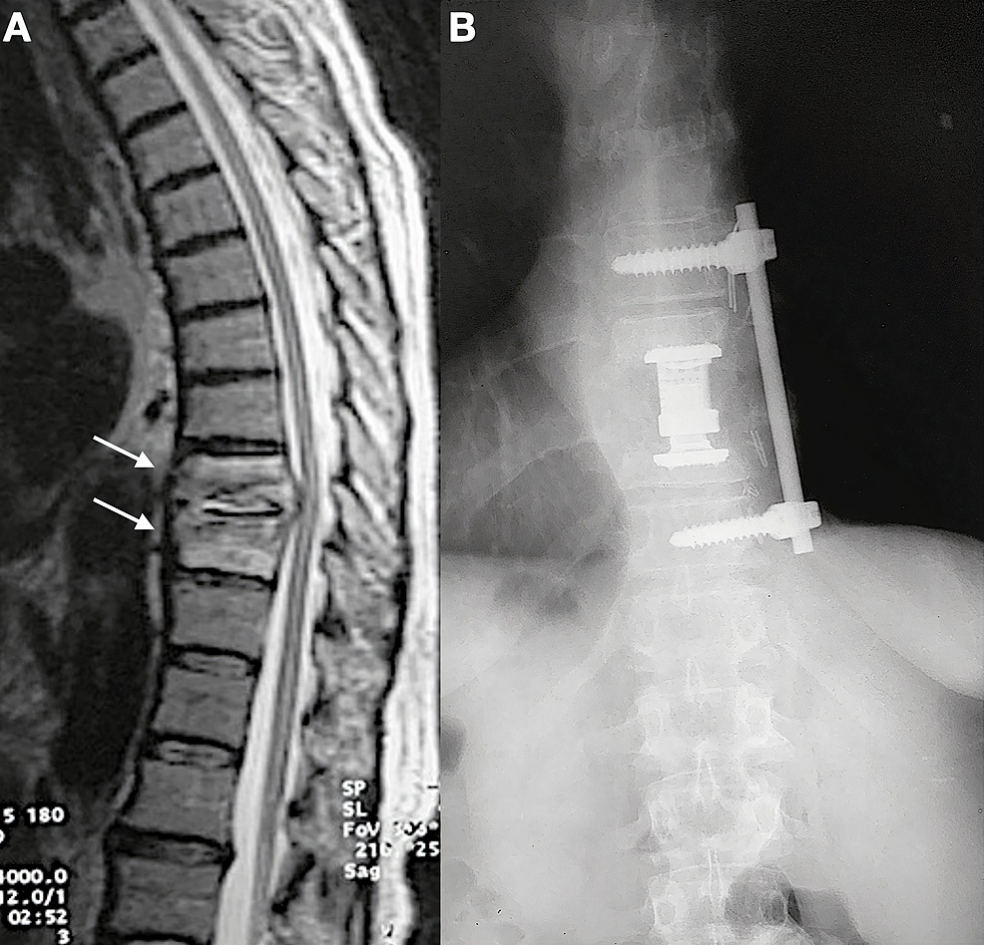
A. Preoperative sagittal T2-weighted image of the lumbar and thoracic spine, showing T10-T11 spondylodiskitis (arrows); B. Postoperative anteroposterior radiograph after the introduction of a titanium mesh cage and anterior instrumentation

**Figure 3:**
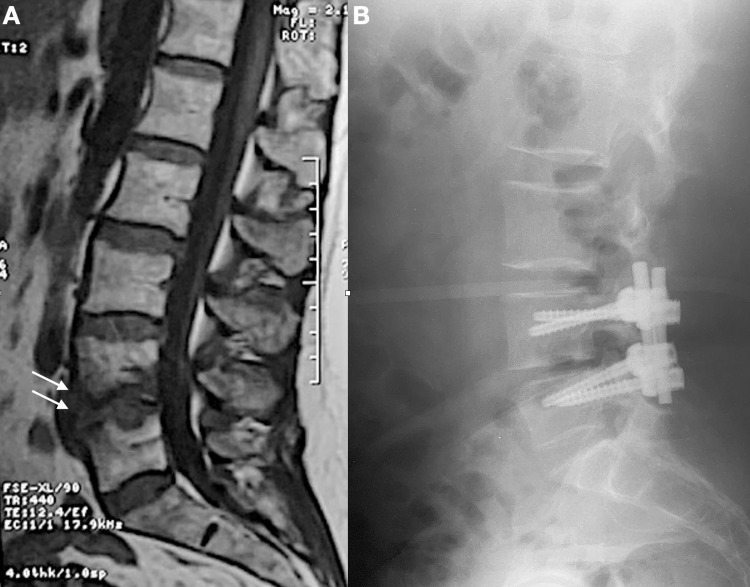
A. Preoperative sagittal T1-weighted image of the lumbar spine, showing L4-L5 spondylodiskitis (arrows); B. Postoperative lateral radiograph after debridement, decompression, and posterior instrumentation

All the patients underwent a one-stage procedure. A combined anterior and posterior approach was used in 28 patients (Figures 1-2). Four patients underwent a posterior approach alone for decompression and stabilization (Figure 3). In all the cases, pus was drained and the inflamed tissue and discs were extensively debrided, followed by decompression, bone grafting from the iliac crest, and interbody fusion. In 20 patients, the anterior column was severely compromised. In those patients, a corpectomy was performed and a titanium cage was used for reconstruction. All the operations were performed by the same surgeon.

Postoperatively, all the patients received intravenous antibiotic treatment according to the resistance spectrum, for at least six weeks, and switched over to oral antibiotics when a clear clinical and laboratory improvement was recorded. Patients received oral antibiotics for a minimum of six weeks. If possible, the patients were mobilized on Day 2 postoperatively.

Outcome measures

The primary outcome measured by this study was healing without residual disability, defined by the absence of clinical symptoms, normalization of CRP and ESR, and absence of active infection in the radiological examinations. Fusion was defined as the presence of a continuous bridging bone between the vertebral bodies, as shown in the sagittal plane. The following data were collected for statistical analysis: age, gender, tissue cultures, operating time, blood loss, complications, and neurological status preoperatively and one year after the operation graded according to American Spinal Injury Association (ASIA) Impairment Scale (AIS).

Statistical analysis

The data were entered into an Excel sheet (Microsoft Corporation, Redmond, Washington) and were analyzed using Statistical Program for Social Sciences (SPSS) 21.0 (IBM Corporation, Armonk, NY). Categorical variables were expressed as frequencies and percentages. Continuous variables were given as mean (range).

## Results

Characteristics of the study population: Microbiology

The study included a total of 32 patients diagnosed with SD and treated operatively. Four patients (12.5%) were treated operatively due to progressive neurologic involvement, four patients (12.5%) due to progressive spinal deformity or instability, two patients (6.3%) due to impending fracture, two patients (6.3%) due to an epidural abscess, and 20 patients (62.5%) due to poor response to antibiotics. There were 18 males (56.2%) and 14 females (43.8%). The mean age of the patients was 68.4 years (range 56-78). SD affected the cervical spine in two cases (6.3%), the thoracic spine in 12 cases (37.5%), and the lumbar spine in 18 cases (56.3%).

The causative microorganism was identified in all cases preoperatively, either by blood cultures (n=11, 34.4%) or by CT-guided biopsies (n=21, 65.6%). The most commonly isolated microorganisms were Staphylococcus aureus (n=18, 56.3%) and Escherichia coli (n=6, 18.6%) followed by Staphylococcus epidermidis (n=3, 9.4%), Mycobacterium tuberculosis (n=2, 6.2%), group B Streptococcus (n=1, 3.1%), Pseudomonas aeruginosa (n=1, 3.1%) and Brucella melitensis (n=1, 3.1%).

The mean operating time was 300 min (range 197-344) for patients that were treated with a combined anterior and posterior approach and 123 min (range 98-189) for the patients that were treated with a posterior approach only. The mean blood loss was 1420 mL (range 1201-1884) and 679 mL (range 540-1021) for the combined and the posterior-only approach, respectively.

Outcome

No mortalities or severe complications were reported. One patient (3.1%) experienced a superficial wound infection that was managed conservatively with topical wound care.

The long-term outcome was favorable for all the patients. All the patients remained disease-free up to the last follow-up (mean follow-up 5.6 years (range 3.5-6.5), without any clinical, radiological, or laboratory sign of relapse or residual infection. Fusion was achieved in all the patients. The mean time of mobilization was 7.3 days (range 2-15). All the patients returned to their regular daily activity by three months. As far as the neurological status is concerned, 27 patients (84.4%) had no neurological impairment preoperatively (ASIA A). Three of them (9.4%) were classified as ASIA C and one of them (3.1%) as ASIA D. At the one-year follow-up, no neurologic deficits were recorded. All the patients were classified as ASIA A.

## Discussion

Although SD is a rare disease, several reports suggest an increase in incidence [11-12] that could be attributed to the aging population and growing rates of drug abuse [13]. Spinal infections are associated with prolonged hospital stays and impose a significant financial burden on the healthcare system [14].

In our study, SD had an increased incidence among men compared to women. The pathogen isolated in more than half of the cases was Staphylococcus aureus (56.3%) and the segment involved most commonly was the lumbar spine. Those results are similar to what has been reported by other studies [7,15-16].

Controversy exists over the duration of conservative treatment before switching to operative treatment. Intravenous antibiotic treatment followed by oral antibiotics, when a clinical and laboratory improvement is noted, remains the mainstay of treatment. Absolute indications of surgical treatment are neurologic deficits, instability, epidural abscess, sepsis, and failure of conservative treatment [17]. Early diagnosis and treatment may be the key to a successful outcome. Previous studies report an improved quality of life and a shorter hospital stay in patients treated operatively as compared to those receiving only antibiotic therapy [18]. In our series, early surgical treatment may be the reason for the complete resolution of neurological deficits recorded preoperatively.

The posterior approach is widely used for the treatment of SD as most surgeons are familiar with this procedure. However, frequently, adequate exposure and debridement of the intervertebral disk and the inflamed tissue are not feasible with a posterior approach. In our study, a combined anterior and posterior approach was used in 28 (87.5%) patients. The role of implants in spinal infection remains uncertain. A single-stage procedure was performed in all the patients of our study and in 20 (62.5%), a titanium mesh cage filled with autogenous bone graft was inserted when a corpectomy was necessary. No implant-related complications were recorded. The infection was eradicated in all the cases and no revision surgery was required. The beneficial role of instrumentation and especially titanium cages is supported by many reports in the literature [19-21].

The present study had some limitations. First, it was a retrospective study and there was no control group of patients. Second, as SD is a rare disease and patients usually receive conservative treatment, the sample of patients was relatively small. Finally, it is stated that the patients of the study received “early” surgical treatment. In the absence of a grading system of SD, it is difficult to draw conclusions as to what “early” surgical treatment really means and how it is associated with improved results.

## Conclusions

In conclusion, our data confirm that when surgical treatment is based on proper indications and is adequately applied, it offers excellent results. In our study, the operative treatment led to a complete resolution of neurological deficits and was associated with a very low complication rate. A single-stage procedure and spinal instrumentation appear to be a safe and effective option when neurologic deficits, instability, sepsis, and epidural abscess are present. Further prospective random control studies are needed to validate the results of our study.
